# Innovations in pediatric imaging: a scoping review of the past decade with case illustrations

**DOI:** 10.1007/s12519-026-01037-2

**Published:** 2026-05-21

**Authors:** Meinrad J. Beer, Matthias C. Schaal, Henning Neubauer

**Affiliations:** 1 Department of Diagnostic and Interventional Radiology, University Hospital Ulm, Albert-Einstein-Allee 23, 89081 Ulm, Germany; 2 SRH Outpatient Clinic of Radiology, 98527 Suhl, Germany

**Keywords:** Artificial intelligence, Innovations, Pediatric imaging

## Abstract

**Background:**

Imaging plays a fundamental and increasing role in the diagnostic work-up of pediatric patients. Non-invasive imaging methods include ultrasonography, X-ray, computed tomography (CT), and magnetic resonance imaging (MRI). The importance of pediatric imaging is reflected in a large, and growing, body of scientific literature.

**Methods:**

A scoping review was conducted following the Preferred Reporting Items for Systematic Reviews and Meta-Analyses guidelines. MEDLINE (PubMed) was systematically searched in February 2025 for English-language original studies published between 2015 and 2025 in peer-reviewed journals. Additionally, illustrative cases from our institutional cohort were included to complement the review findings.

**Results:**

The presented scoping review demonstrates significant technical innovations in all explored imaging modalities. In ultrasonography, trauma imaging and contrast-enhanced ultrasound beyond voiding ultrasound are major areas of progress. X-ray and CT imaging in children benefit from improved radiation protection. Photon-counting CT has been introduced into routine clinical practice. Low-field, real-time, and CT-like MRI reduces the need for sedation and makes MRI faster, safer, and more accurate while opening new application possibilities. Finally, improvements in hybrid imaging [positron emission tomography (PET)/CT and PET/MRI], new contrast agents, and the implementation of advanced artificial intelligence techniques are pushing boundaries in diagnostic imaging.

**Conclusion:**

A decade of on-going technical innovation has provided interesting new tools for safer, faster, and more patient-centered clinical imaging studies in children and adolescents.

**Graphical abstract:**

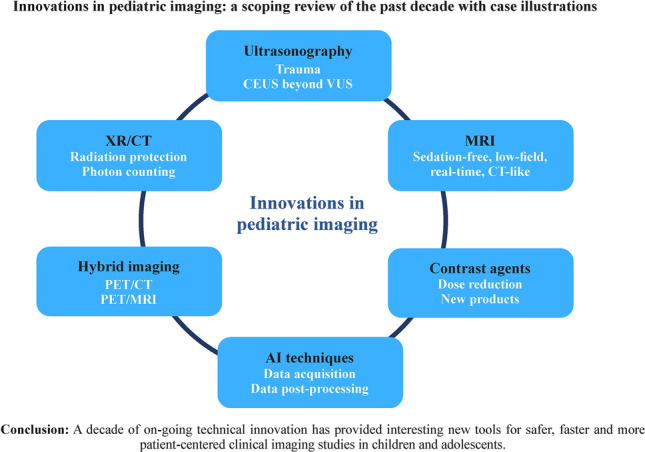

## Introduction

Non-invasive imaging has played a fundamental and increasing role in pediatric and adolescent diseases. Typically, non-invasive imaging encompasses ultrasound, radiography, computed tomography (CT), and magnetic resonance imaging (MRI). The increasing importance of diagnostic imaging in clinical practice is supported by a large, and continuously growing, body of scientific literature. PubMed contains almost 10,000 publications under the keyword “advances in pediatric imaging” and more than 3000 publications under the keyword “innovations in pediatric imaging”. However, most of these studies are highly specialized publications that focus on small cohorts of pediatric patients with specific diseases and often cannot be directly translated into routine clinical imaging. We, therefore, perceive a need for a general overview of established and emerging imaging techniques for children and adolescents with a special focus on innovation.

The format of a scoping review following the Preferred Reporting Items for Systematic Reviews and Meta-Analyses concept was adopted for this endeavor, focusing on studies published in peer-reviewed journals over the last 10 years, enriched by case series from our own cohort as an illustration to the review [1].

## Methods

### Search strategy

A systematic search of studies in the English language was performed on MEDLINE in February 2025 (PubMed, https://www.ncbi.nlm.nih.gov/pubmed/). The database query was limited to original human studies in peer-reviewed journals with an available abstract. For the publication date, the limits “all” and “the last 10 years” (2015–2025) were applied.

Our aim was to identify studies assessing the value of routinely used imaging methods, such as plain radiography, ultrasound, CT, MRI, and hybrid imaging, plus relevant topics, such as contrast agent and artificial intelligence (AI) applications. We used the terms “advances in pediatric imaging” and “innovations in pediatric imaging”, the former including studies that analyze the value of improvements in existing technologies and the latter encompassing studies that introduce and apply new and innovative technologies.

The inclusion criteria were as follows: (1) original research; (2) sufficiently large patient groups (*n* ≥ 10); and (3) focused on a pediatric population.

### Secondary targeted search

Initially, the search strings produced many entries. A first assessment demonstrated, as stated above, that most articles were highly specific. Thus, a second database query was performed with more detailed search strings.

All three authors (each with more than 10 years of experience in pediatric imaging) specified search strings according to hot topics in international meetings or conferences, grouped for the different imaging modalities. The following search terms were used: “plain radiography dose reduction children”, “ultrasound application fracture detection children”, “contrast-enhanced ultrasound (CEUS) applications children”, “CT applications trauma children protection”, “photon-counting CT (PCCT) children”, “MRI minimizing sedation children”, “ultrafast MRI children”, “low-field MRI applications children”, “synthetic CT images MRI children”, “advances positron emission tomography (PET) MRI applications children”, “contrast-media iodine safety and risks children”, “new contrast agent MRI macrocyclic children”, “AI-based image acquisition children”, “AI-based image postprocessing children”, and “AI image postprocessing children”.

### Study selection

In the next step, after the removal of duplicates, only titles and abstracts were screened. All the articles that did not meet the inclusion criteria were excluded from further analysis. The remaining articles were chosen for reading the full text. Finally, the references of the included articles were manually searched to check for further eligible studies.

### Illustrative cases from clinical practice

To illustrate the striking innovations in pediatric imaging techniques in recent years, five clinical case scenarios have been chosen, with a focus on ultrasound, CT and MRI/hybrid imaging. All case illustrations were derived from anonymized patient data within ethically approved institutional research.

## Results

### Literature search

**Fig. 1:**
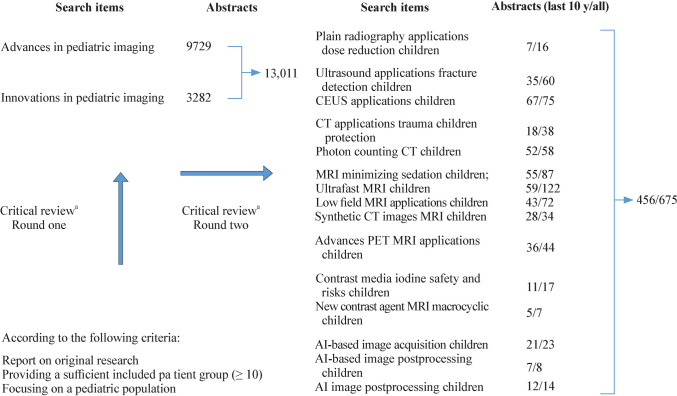
Flow diagram of the search strategy for PubMed. The critical review was performed in two rounds according to the following criteria: reporting on original research; providing a sufficient included patient group (≥ 10); and focusing on a pediatric population. *CT* computed tomography, *MRI* magnetic resonance imaging, *AI* artificial intelligence, *CEUS* contrast-enhanced ultrasound, *PET* positron emission tomography. ^a^By readers with experience of several years in pediatric imaging

Initially, the search strings produced the following number of entries: 9729 with “advances in pediatric imaging” and 3282 with “innovations in pediatric imaging”. The second targeted search yielded 456 entries (last 10 years) and 675 entries (all years) across all the modalities. The distribution of the search results is presented in Fig. 1.

### Plain radiography

The main focus of plain radiography applications in children has been, and still is, dose reduction. Abdominal examinations, in particular, involve significant amounts of ionizing radiation, whereby the skin entrance dose is an important co-factor and dose levels might be higher in adolescents than previously reported [2]. Simple technical precautions, such as adjusting the exposure class, lead to significant reductions and thus the optimization of dose levels [3].

### Ultrasound

With respect to ultrasonography, three main focal points could be identified as arising topics over the past 10 years: trauma/emergency imaging, contrast-enhanced imaging of the vesico-urinary tract [voiding urosonography (VUS)], and intravenous CEUS.

For trauma imaging, ultrasonography is becoming increasingly feasible for the assessment of peripheral bone fractures. New national guidelines suggest that sonography is diagnostically effective in the primary assessment of suspected peripheral bone fractures of the upper extremity. The BUCKLED study demonstrated the benefit of sonography-based fracture exclusion diagnostics in the lower distal extremities [4]. The elbow joint poses a particular challenge in pediatric trauma diagnostics. This makes the review by Averill et al. [5] on ultrasound examinations of the child’s elbow all the more important. A standardized performance and documentation of the examination is essential to ensure transparency and traceability.

VUS is increasingly frequently used for the detection of vesicoureteral reflux (VUR) in daily clinical practice. Recently, new consensus recommendations were published by the European Society of Pediatric Radiology and its Working Group on Uroradiology [6]. Since interdisciplinary treatment is necessary in the case of VUR, structured reporting of voiding cystourethrography (VCUG) via X-ray and fluoroscopic imaging and VUS is important. A corresponding template has been developed. The advantages of the VUS technique over the VCUG examination are also presented. In children and adolescents, CEUS after intravenous application of a contrast agent is increasingly used for imaging solid organ systems, such as the liver, spleen, and lymph nodes [7]. Its safety has been proven [8], although the off-label use required in various countries poses special challenges in the arena of pediatric radiology and beyond [9].

### Computed tomography

The use of CT in the management of pediatric trauma has been discussed intensively in recent years, with a special focus on radiation protection by shielding. PCCT is another hot topic in pediatric imaging, as it has the potential to significantly improve spatial resolution plus significantly reduce scanning time and radiation exposure.

Imaging of polytrauma using whole-body CT is routinely performed in children and adolescents. The associated radiation exposure is the subject of controversial debate. Didion et al. investigated injury patterns, the use of CT protocols, and the role of “second readings” for polytrauma CT examinations in a large group of more than 1000 children and adolescents in 63 imaging centers over a period of 9 years [10]. Almost two-thirds of the children and adolescents had multiple injuries, and a second reading led to the detection of additional injuries in 8% of the patients. Among these children and adolescents with polytrauma, slightly more than 1% had an active hemorrhage or a pseudoaneurysm. This publication highlights the importance of polytrauma CT in pediatric trauma patients and suggests that at least multiphase CT scans may not be necessary in pediatric patients, as the majority of injuries can be detected even without contrast agent administration.

As described above, radiation exposure is viewed critically, particularly in pediatric CT examinations. In this respect, the use of radiation protection devices is important and is currently being discussed. Granata et al. presented the results of a large European study, which revealed that the use of primary (filters, etc.) and secondary radiation (lead shielding, etc.) protection varies widely between imaging centers [11]. This applies to both adults and children. On CT, radiation protection within the radiation field is used in about one-fifth of patients. In one-third of patients, radiation protection, especially gonadal protection, is used outside the field. Another question is whether modern CT technology has more advantages than disadvantages. This phenomenon was investigated by Larjava et al. [12]. Phantom studies have shown that “overscanning” leads to relevant increases in radiation dose. An increase in dose occurred several centimeters before the end of the scanning path. Highly significant dose increases occurred during the last shift when radiation protection agents were used. Compared with conventional (energy-integrating) CT technology, PCCT offers numerous advantages, particularly in terms of dose, image quality, and spectral informative value. The first publications on PCCT technology over the last two years demonstrated its particular value in pediatric patients. Following initial feasibility studies, the first systematic comparative analyses were recently published [13]. In a direct 1-to-1 comparison, significantly less radiation was administered in PCCT, with improved image quality and alleviated motion artifacts. This was shown not only for lung imaging but also for PCCT of other body parts, such as abdominopelvic imaging [14].

### Magnetic resonance imaging

The child-centered management of pediatric MR images (preparation, mock scanner, and ambient light) is one of the hot topics in current research, along with low-field MRI, rapid MRI, and synthetic CT-like MRI. Fletcher et al. investigated the effectiveness of different training approaches for performing MRI examinations in children without sedation, if possible [15]. Three approaches were compared and analyzed with regard to their effectiveness and acceptance rates by both children and accompanying parents: (1) preparation for the examination at home by providing videos with and without sounds as well as book-based illustrative material; (2) the presence of a psychologist on the spot during the MRI examinations; and (3) preparation using a miniature/play MRI, a so-called “mock scanner”. All three approaches significantly improved patient handling and thus reduced the need for sedation. Over 90% of the children were able to undergo MRI without sedation. There were no significant differences among the three approaches. Surprisingly, a trend toward better results was observed with the approach of preparing at home using visual material.

The use of so-called low-field MRI technologies in children and adolescents has increased in speed again in the past year. The advantages here are the easy examination conditions, which require little preparation on the part of the children and adolescents as well as the accompanying parents. Owing to AI support, the image quality of these low-field systems is now comparable to that of older examinations at 1.5 Tesla. While the first low-field examinations focused on applications in the area of the brain, further publications on the use of low-field technology in musculoskeletal or abdominal imaging have been published in the past 12 months. A study by the University of Michigan, which examined a total of nearly 60 children, reported that the accuracy of low-field 0.5 Tesla MRI is comparable to that of 1.5 Tesla MRI in abdominal imaging studies [16].

Heiss et al. performed both morphological and functional lung MRI examinations in children with a history of COVID-19 infection [17, 18]. In a cohort of 54 children, functional limitations can be detected more than 12 months post-infection. Low-field MRI allows good regional assessment of lung function. New technical approaches feature real-time MRI. Hirsch et al. presented the first results of “real-time” MRI [19]. This technology, known as “Flash 2.0”, requires a manufacturer’s 3.0 Tesla platform with additional local data postprocessing. Irrespective of this current limitation, significantly better localization of at least larger pulmonary lesions is possible in a very short time (12–19 seconds). However, such scans cannot yet serve as a substitute for CT studies, as the detection limit is 10 mm in lesion size, meaning that small metastases cannot be detected with sufficient sensitivity.

The establishment and clinical evaluation of synthetic CT-like images based on modern MRI sequences has been a very important advance in the field of musculoskeletal imaging in children and adolescents over the past several years. Novel MRI sequences with very short echo times (TEs) have been developed and dubbed ultrashort/zero-TE imaging (UTE/ZTE). Head-and-neck imaging, in particular, benefits from that technology [20]. Synthetic CT-like image generation using UTE-MRI is possible in everyday practice and shows very good agreement with CT imaging. However, it has also been shown that MRI-based synthetic CT-like imaging produces equivalent results to conventional CT imaging in other regions of the body, particularly in the iliosacral joints [21]. UTE/ZTE imaging of other large joints, e.g., the hip joint, is also feasible and produces imaging results comparable to those of real-world CT studies [22]. In this study, Iwasaka-Neder et al. used 3 Tesla MRI to generate CT-like MR images in a rather small group of children and adolescents (*n* = 19) with excellent agreement between CT and MRI scans, resulting in an accuracy of up to 98%.

Lung imaging also significantly benefits from UTE/ZTE technologies. 3D UTE-MRI offers a sensitive method for regional volumetric evaluation in chronic lung disease, such as cystic fibrosis, and is much more accessible than current state-of-the-art technologies, such as Xe-MRI [23]. In addition to lung imaging, ZTE MRI is a potential emerging non-irradiating method for the diagnosis and management of urolithiasis in children, with accuracies of 81.8% and 93.7% for stones larger than 5 mm and 10 mm, respectively [24]. Additionally, imaging of pelvic inflammation is improved by ZTE MRI, providing sacroiliac joint visualization comparable to that of low-dose CT scans, thereby allowing the detection of subtle erosions and sclerosis in these joints [25].

### Hybrid imaging

Hybrid imaging techniques, such as PET/CT and especially PET/MRI, play a central role in pediatric oncology, as they combine anatomical and metabolic information and allow multiparametric assessment with high sensitivity in primary diagnosis, differential diagnosis, and follow-up [26]. In recent years, significant improvements have been made in optimizing sequences and postprocessing workflows [27]. Owing to its lower combined radiation dose, PET/MRI has advantages compared with PET/CT, with superiority concerning anatomical information [28, 29]. Improvements in the field of nuclear medicine examinations in children and adolescents, especially those with tumors, have been made with respect to radiation exposure by reducing the administered dose of radiopharmaceuticals [30].

### Contrast media

Contrast-enhanced imaging is typically performed in pediatric radiology. In some cases, there is uncertainty regarding a specific risk profile with regard to the administration of contrast media. Cheeney et al. compiled an overview of ultrasound, CT, and MRI contrast agents with respect to characteristics, application rates, complication rates, and measures in the event of an adverse reaction [31]. Although this overview focuses on the USA, it shows that contrast media can generally be used in children and adolescents with a very low-risk profile. The overall complication rates are low.

The relationship between acute kidney injury and previous iodine-based contrast agent administration for CT examinations was investigated by Calle-Toro et al. in more than 10,000 children and adolescents [32]. The risk was found to be even lower than that reported in the previous publications, averaging 1.5%. However, this study was exclusively performed in children and adolescents with a reduced glomerular filtration rate of less than 60 mL/min/1.73 m^2^. The only limitation in this retrospective analysis was the moderately long follow-up period of 84 hours, which calls for further studies to address the possible impact of lead time on the study results.

In recent years, the MRI contrast agents used in children have been almost exclusively of the macrocyclic structural type, thus limiting the risk of gadolinium release from the contrast agent and its subsequent deposition and accumulation in body tissues. Currently, a new class of MRI contrast agent containing less gadolinium per volume has become available for adult and pediatric patients. The first reports demonstrated a high safety profile in combination with improved image quality in different body regions [33, 34].

### Artificial intelligence

Data acquisition and data postprocessing benefit largely from AI techniques. AI-based sequence acceleration is a major breakthrough in MRI. In addition to ultrasound, MRI is the main imaging modality in pediatric radiology, even though a long acquisition time has always been a challenge in young patients. AI-based techniques have the potential to significantly reduce MRI scan times. Kim et al. investigated the use of deep-learning-based accelerated MR neuroimaging in everyday clinical practice [35]. Three imaging approaches were compared: a standard protocol, an accelerated protocol, and a deep-learning-based image reconstruction of the accelerated protocol. Compared with traditional reconstruction, AI-based image reconstruction can reduce the acquisition time by up to 42% while maintaining image quality and keeping the T1, T2, and PD contrasts largely unchanged. Notably, this was a synthetic multicontrast sequence that combined four image sequences (T2, fluid-attenuated inversion recovery; T1; and phase-sensitive inversion recovery); in addition, 5 of the 47 examinations were excluded because of artifacts, so there is still scope for further research and development.

AI-based improvements in data postprocessing have also been demonstrated in thoracic and musculoskeletal imaging. Schalekamp et al. summarized the current status [36]. Interestingly, more than 40 certified software applications were already available in 2022. However, these algorithms were almost exclusively trained for adult patients. However, algorithms for the clinically important oncological diagnosis of round lung lesions are still lacking. In view of the rapid pace of development, however, many software solutions can be expected in the future. The challenge here is that children and adolescents have very different thorax geometries depending on their age, which makes training AI-based algorithms difficult. In addition, the area of application is smaller than that of adult radiology.

First, commercial AI applications are available for pediatric conventional radiological trauma diagnostics. Like thoracic diagnostics, these methods are limited by a mostly non-specific pediatric radiological approach. Zech et al. developed a specific AI algorithm for fractures of the upper extremity with high discriminatory power [37]. The results of the AI algorithm were more accurate than those of the radiology residents. AI-based automatic data analysis or aggregation might also have utility for imaging suspected neonatal hypoxia. In the so-called HEAL trial, Calabrese and coauthors examined the results of a large cohort of over 400 newborns with peripartum hypoxia [38]. The performance of an MRI, including diffusion-weighted imaging, plays an essential role here. During a follow-up period of 2 years, acute brain damage was found in just under one-third (27%) of the more than 400 children, most of whom were born mature, with a mean gestational age of 39 weeks. This could be determined both on the basis of the affected volume of the entire brain and, in particular, on the basis of the findings on diffusion-weighted imaging. The determination of the affected brain volume and the apparent diffusion coefficient area can be automated using AI-based algorithms. The volumes measured were strongly correlated with morbidity and, in particular, mortality. This means that a new quantitative parameter is now available that can replace existing qualitative parameters in the future. In addition to neuroimaging, AI techniques are increasingly integrated into multiparametric oncologic imaging [39].

### Illustrative cases

**Fig. 2:**
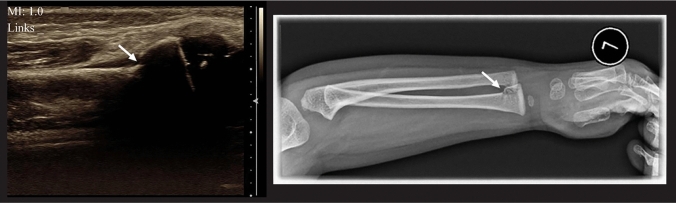
Detection of a distal fracture of the upper extremities by ultrasound compared with X-ray: a 2-year-old boy after a fall on his left wrist, clinically suspected fracture. Ultrasound (left side) is comparable to plain radiography (right side) in terms of the discontinuation of cortical bone (arrow) and the shift in the bone axis

**Fig. 3:**
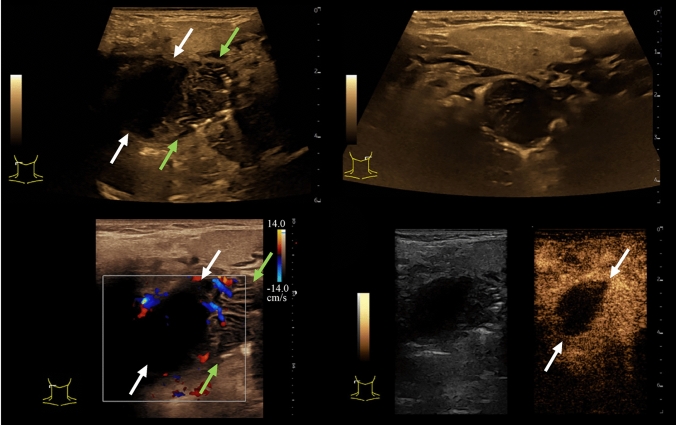
Detection of a tonsillar abscess via contrast-enhanced ultrasound (CEUS): a 10-year-old girl with a clinically suspected right-sided peritonsillar abscess. Ultrasound in B-mode (left upper) and color-coded duplex mode (left lower) revealed a hypoechogenic formation suspicious of abscess formation (white arrows in left upper and left lower) laterally to the tonsil (green arrows in left upper and left lower) for comparison with the non-affected left side in B-mode (right upper). CEUS after intravenous weight-adapted application of contrast medium clearly confirmed the lack of perfusion of the abscess (white arrows at the lower right)

The first clinical vignette involves a toddler with a distal radius fracture of the left arm. Ultrasound depicts the discontinuation of cortical bone and the shift of the bone axis, comparable to plain radiography (Fig. 2). A melting tonsillar abscess is a challenge in pediatric imaging. Ultrasound allows fast bedside assessment of the tonsillar region, which can be depicted below the submandibular gland. However, the sensitivity for necrosis/melting is limited with ultrasonography. In this setting, CEUS allows a highly sensitive and specific evaluation. Figure 3 shows the ultrasound and CEUS findings of a melting tonsillar abscess in the right tonsillar recess of a child. Grayscale ultrasound revealed an enlarged right tonsillar region with areas of low echogenicity—especially in comparison to the non-diseased left side. When CEUS is added, the abscess on the affected side is clearly depicted.

**Fig. 4:**
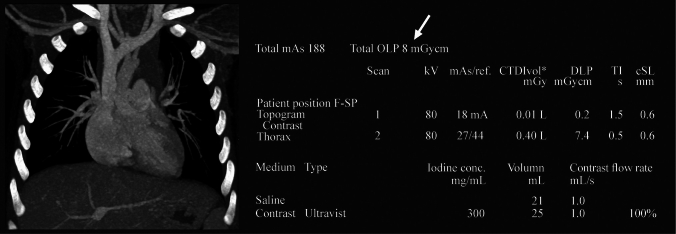
Ultralow-dose contrast-enhanced images of the thorax with an optimized radiation dose: contrast-enhanced computed tomography examination in the arterial-venous mixed phase of an infant to exclude prenatally suspected sequestration and a maximum intensity projection on the left side in the coronal reconstruction. An ultralow-dose protocol was applied with a dose-length product of less than 10 mGycm (arrow on the right side). Contrast agent was applied by hand; for additional reduction in the radiation dose, no bolus tracking was used

Minimizing the radiation dose is a major concern in pediatric CT imaging. Typically, CT usage is restricted to musculoskeletal imaging and non-contrast low-dose lung/thoracic imaging. In specific scenarios, contrast-enhanced thoracic CT is applied, for example, to evaluate suspected vascular anomalies, which might also have an impact on the lung parenchyma. Sequestration leads to hyperperfusion, with implications for the lung parenchyma. Figure 4 presents a contrast-enhanced CT image of an infant with the purpose of excluding suspected sequestration. An ultralow-dose protocol was applied with a dose-length product of less than 10 mGycm.

**Fig. 5:**
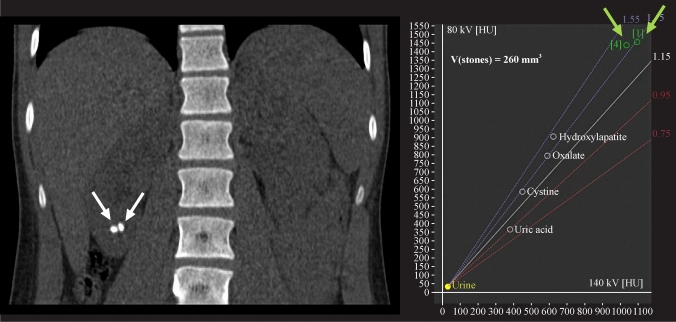
Characterization of kidney stones via spectral (dual-energy) computed tomography (CT): two kidney stones in a 14-year-old girl who was sonographically diagnosed with nephrolithiasis. CT (dual-energy) was performed for stone analysis, which detected two renal concretions in the lower pole of the right kidney (arrows on the right side). To reduce the radiation dose, the scan length was limited to the kidney region. On the basis of the dual-energy analysis, one of the stones was most likely an oxalate stone, and the other was a mixed oxalate/hydroxyapatite stone (green arrows on the right side)

Spectral CT based on dual-energy techniques allows for material decomposition and thus the characterization of kidney stones. Figure 5 shows two kidney stones in the lower right kidney of an adolescent. Dual-source spectral-CT imaging characterizes these renal concretions as one oxalate stone and one hydroxyapatite stone.

**Fig. 6:**
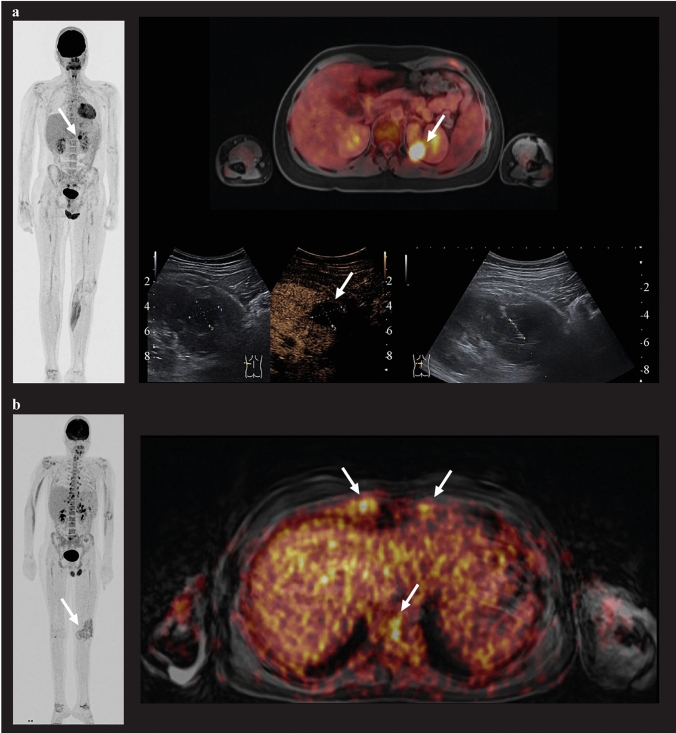
Multiparametric hybrid imaging for characterization of multiorgan fungal infection in a 15-year-old immunocompromised male adolescent (with myelodysplastic syndrome after stem cell transplantation) with clinical suspicion of fungal sepsis. **a** Whole-body PET/MRI with Fluor-18-fluorodeoxyglucose was performed to locate the inflammatory focus (left side: MIP and right upper: fused anatomical image in axial orientation), with an intense hypermetabolic focus at the upper pole of the left kidney (arrows). An ultrasound-guided biopsy of the left kidney was performed (after confirmation of localization by CEUS, arrow right lower); unfortunately, no pathogen could be detected in the biopsy; **b** despite anti-inflammatory and antibacterial/antifungal therapies, the clinical status of the patient deteriorated. Therefore, a follow-up PET/MRI was performed, which revealed additional uptake in several bones (right side: fused anatomical image in the axial orientation with arrows marking hypermetabolic foci in the vertebral body and costovertebral joints and image blurring due to pain-related movement artifacts). A puncture of the left knee joint with hypermetabolic metabolism (arrow in the MIP on the left side) confirmed the diagnosis of an infection with fusaria. A specific antifungal therapy was effective in curbing and eliminating the infection. *PET* positron emission tomography, *MRI* magnetic resonance imaging, *CEUS* contrast-enhanced ultrasound, *MIP* maximum intensity projection

Hybrid imaging is the ideal tool for assessing whole-body disease in oncology and infections. Figure 6 summarizes the PET-MRI findings in an immunocompromised adolescent with Fusarium infiltrates who subsequently underwent an ultrasound-guided biopsy of a kidney area. In the first PET-MRI scan, a focus of increased metabolism is depicted in the left kidney, with additional diffuse uptake in one part of the muscles of the upper left lower extremity attributed to muscle activity. An ultrasound-guided biopsy of the left kidney was performed, but unfortunately, no pathogen could be detected in the biopsy. Despite anti-inflammatory and antibacterial/fungal therapy, the clinical status of the patient has deteriorated. A follow-up PET-MRI scan two months later demonstrated additional uptake in bones (especially the vertebral column), enlarged central and inguinal lymph nodes and the left knee. A diagnostic puncture of the left knee joint revealed an infection with Fusarium. Specific antifungal therapy eventually stopped and eliminated the infection.

## Discussion

The graphical abstract summarizes the main innovations and their subtopics in pediatric imaging modalities over the past 10 years. We focused on the past decade, as many of the most important innovations discussed in this text have become available in clinical practice only recently. Another important aspect of innovation is the finding that innovation now extends beyond hardware and software to encompass the entire patient journey, from previsit preparation materials to postprocedure follow-up, consequently resulting in a reduced need for sedation in pediatric MRI studies, as an example. Therefore, the pediatric imaging landscape is currently undergoing cultural transformation, with the recognition that technical excellence must be coupled with awareness.

Despite the endeavor to provide a comprehensive overview, this review has limitations. First, a language bias, which relies on the structure of the applied search machines such as PubMed, may exist. However, an increasing number of publications originating from non-English-speaking countries are now published in two languages, in the national language and in English. This helps reduce the limiting factor of potentially neglecting non-English native publications. Second, a general lack of validation for AI tools in pediatric populations is worth mentioning, along with the well-known general fairness gap of many AI tools, mostly in the areas of sex and age [40]. An intraclass coefficient analysis during the data selection process may have improved the process; however, this was not carried out. With respect to technological improvements in hardware and software, such as low-field MRI, these improvements are readily available to the same extent across institutions or even countries. Nevertheless, these limitations also open the field for future studies, which could focus on multicenter validation of emerging technologies, such as AI and low-field MRI, in pediatric cohorts.

Hereinafter, we present a case series from our own patient cohort to illustrate and highlight the clinical importance of some of the innovations reviewed above. We focus on key elements, such as fracture detection by ultrasound, abscess detection by CEUS, dose reduction by ultralow-dose contrast-enhanced CT, urinary tract stone characterization by spectral-CT and multiparametric analysis by PET/MRI as a hybrid imaging technology.

The current scoping review, with a temporal focus on the last decade, demonstrates significant innovations across all studied imaging modalities. Our review is original and stands out from other works in that it comprehensively addresses all relevant pediatric radiological imaging methods, highlights the evolutionary and revolutionary innovations of the last 10 years, and vividly illustrates their real clinical value via practical examples from our own patient cohort. The case series presented here was selected from clinical routine examinations at our institution to highlight the immediate clinical applicability of these new imaging procedures, provided that the necessary technical equipment and trained radiologists are available.

In conclusion, all the presented innovations open up a multitude of new choices and opportunities for safer, faster and more accurate imaging procedures and, eventually, better clinical outcomes in pediatric patients.

## Data Availability

The datasets generated and/or analyzed during the current study are available from the corresponding author on reasonable request.
